# Exploring the role of urinary extracellular vesicles in kidney physiology, aging, and disease progression

**DOI:** 10.1152/ajpcell.00349.2023

**Published:** 2023-10-16

**Authors:** Cristina Grange, Alessia Dalmasso, Judiel John Cortez, Beatrice Spokeviciute, Benedetta Bussolati

**Affiliations:** ^1^Department of Medical Sciences, University of Turin, Turin, Italy; ^2^Department of Molecular Biotechnology and Health Sciences, University of Turin, Turin, Italy

**Keywords:** EV release, exosomes, renal injury, renal tubular cells, urine

## Abstract

Extracellular vesicles (EVs), membranous vesicles present in all body fluids, are considered important messengers, carrying their information over long distance and modulating the gene expression profile of recipient cells. EVs collected in urine (uEVs) are mainly originated from the apical part of urogenital tract, following the urine flow. Moreover, bacterial-derived EVs are present within urine and may reflect the composition of microbiota. Consolidated evidence has established the involvement of uEVs in renal physiology, being responsible for glomerular and tubular cross talk and among different tubular segments. uEVs may also be involved in other physiological functions such as modulation of innate immunity, coagulation, or metabolic activities. Furthermore, it has been recently remonstrated that age, sex, endurance excise, and lifestyle may influence uEV composition and release, modifying their cargo. On the other hand, uEVs appear modulators of different urogenital pathological conditions, triggering disease progression. uEVs sustain fibrosis and inflammation processes, both involved in acute and chronic kidney diseases, aging, and stone formation. The molecular signature of uEVs collected from diseased patients can be of interest for understanding kidney physiopathology and for identifying diagnostic and prognostic biomarkers.

## Graphical abstract

**Figure:**
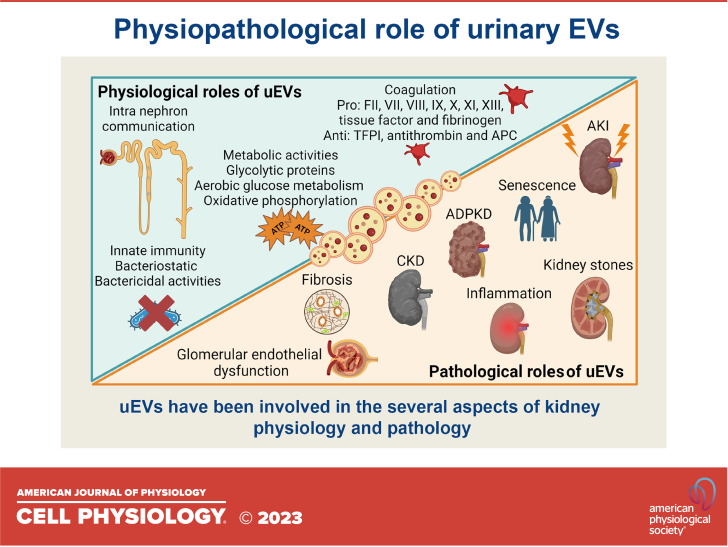

## INTRODUCTION

Kidneys are in control of several fundamental functions of our body as they maintain blood homeostasis, pressure, and composition. In particular, they regulate ion balance and calcium levels, pH, water excretion, clearance and reabsorption of waste products and metabolites, as well as red blood cell number. Kidneys produce ∼1–2 L of urine per day, in which are collected waste products, cells of epithelial or blood origin, viruses, bacteria, soluble molecules, and extracellular vesicles (EVs) (1), small vesicles considered mediators of cell-to-cell communication. Indeed, EVs present in urine appear to play a role as messengers along nephron segments, following the natural flow of preurine and urine. In this review, we will provide a focus on the modulation of EV release in urine and on their role in renal physiology, including glomerular/tubular and tubular/tubular cross talk, and in renal pathology, including aging, stone formation, and disease progression.

## EXTRACELLULAR VESICLES

EVs are a heterogeneous population of small vesicles secreted by almost all cell types in the extracellular space and present in almost all body fluids (2, 3). EVs are composed by a core containing nucleic acids (mRNAs, miRNAs, and other noncoding RNAs), proteins and lipids, derived from the originating cells, surrounded by a lipid bilayer membrane, with a protective function for the EV cargo (2). DNA packaging into EVs has been mainly described in the oncology field, showing EV-DNA uptake and incorporation into the recipient genome (4). On the EV surface, there are several different classes of transmembrane and external proteins that regulate EV biodistribution and targeting ability. When EVs are dispersed within a fluid, the close interaction between EVs and soluble factors leads to the formation of an additional layer, mainly composed by proteins, called protein corona (5). EVs, thanks to their round shape, small size, and their specific fingerprint, appear ideal messengers to deliver their cargo along body fluids, being suitable for therapeutic and diagnostic potential applications.

**Figure 1.**
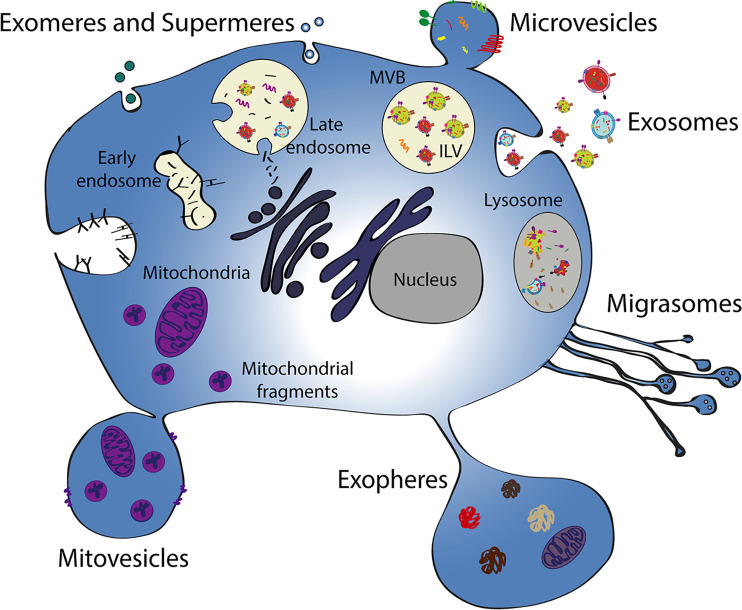
Schematic representation of different EV subtypes and their origin. Different processes of EV generation are schematized, including EV released by budding of the plasma membrane (microvesicles, exopheres, and mitovesicles), by retraction fibers of migrating cells (migrasomes), or by membrane fusion of multivesicular bodies (exosomes). The intracellular steps of endosome maturation and multivesicular body formation and the release of small particles (exomeres and supermeres) are also shown. ILV, intraluminal vesicles; MVB, multivesicular body.

The different EV types can be classified into different subcategories, considering size, density, biogenesis, or isolation strategy, although there is the lack of a unique phenotype for each of them (3). In addition, the different EV subtypes show overlapping properties in terms of size, marker expression, composition, and functions, and there are constant updates in the nomenclature due to scientific advances. The site of biogenesis clearly distinguishes exosomes, arising from inward budding of the endosomal membrane from ectosomes, originating from outward budding of the plasma membrane (3) (Fig. 1). Moreover, there is general consensus in the scientific community to divide EVs based on their size, using small EVs for those ranging from 50 to 150 nm and large EVs for those ranging from 200 nm to around 5 µm; the latter including both microvesicles, ranging from 200 to 1,000 nm, exopheres and apoptotic bodies (with a diameter up to 5 µm) (6). EV subtypes have been also classified by content (such as mitovesicles), function (such as migrasomes), and originating cell (such as oncosomes) (Fig. 1) (3, 7). Furthermore, small particles with a size of around 20–40 nm, called exomeres and supermeres, have been recently identified, even though, in the absence of a double membrane, they do not fit in the EV definition (Fig. 1) (7). Biogenesis and characteristics of the most recently described particles are still under investigation and further study will help to better understand their biology.

Both EV physical/chemical properties and surface markers may affect EVs’ trafficking and uptake by downstream cells. Several mechanisms may occur at the same time, including phagocytosis, plasmatic, or endosomal fusion of membranes, clathrin or caveolin-mediated endocytosis, and macro- and micropinocytosis (8). These topics are nicely summarized in a recent review (3).

Generally, surface and luminal markers shared by EV types are commonly used for EV characterization, including tetraspanins (CD9, CD63, and CD81), surface proteins that organize membrane microdomains and are involved in EV biogenesis process, heat shock protein 70 (HSP70), a chaperone protein, ALG-2-interacting protein X (Alix) and tumor susceptibility gene 101 (TSG101) [auxiliary proteins involved in the endosomal sorting complexes required for transport (ESCRT) pathway] (9). EV cargo was also extensively characterized by adopting bioinformatic methods and reported in comprehensive databases such as ExoCarta, EVpedia, and Vesiclepedia that elucidate molecular content of EVs isolated in physiological or pathological conditions (10). In addition, dedicated databases such as EVatlas or exoRBase describe all the RNA species present in EVs (11).

EV characterization and biomarker identification are affected by the isolation procedures used to collect EVs. This is, at present, a critical point for both comprehensive EV characterization and further clinical applications. In fact, there is no standardized protocol and methodology to isolate pure EV populations, as its choice depends on the downstream analyses and use. Several techniques are available including serial ultracentrifugations, size exclusion chromatography, filtration, precipitation, density-gradient centrifugation, and immunoprecipitation/affinity capture (1).

## ORIGIN OF URINARY EVs

EVs collected into the urine (uEVs) originate from all the cells of the urogenital tract, with the majority of them of renal, prostate, and bladder origin (1). In particular, it has been suggested that the kidney is the main source of uEVs (12). Recently, Barreiro and colleagues recapitulated the uEV composition by an in vitro approach, isolating EVs from proximal tubular, mesangial, podocyte, and glomerular cells. miRNAs, mRNAs, and proteins of uEVs isolated in vitro were compared with those obtained in uEVs, confirming the kidney as major source of uEVs and dissecting single cell-type contribution (13).

The passage of serum EVs into urine may occur through peritubular capillaries, but this phenomenon in vivo has not been clearly demonstrated yet. However, it has been reported that exogenous intravenous administration of PKH67-labeled EVs resulted in the urinary excretion of fluorescent EVs in an experimental in vivo model of acute myocardial infarction (14). It was reported that this phenomenon may be influenced by hormonal regulation, for example, by a vasopressin analog, that increased the renal uptake of systemically injected fluorescent EVs, labeled with cell tracker nanocrystals (15). These results suggest the presence of an excretion into the urine of not-urinary EVs; however, in physiological conditions, this phenomenon has not been confirmed, and this trafficking is still unclear (16). Using a microfluidic model composed by a double layer of cells mimicking the glomerular barrier, Bellucci et al. (17) recently demonstrated that EVs, engineered with nonhuman cel-miR-39, were able to cross the glomerular barrier and to transfer the miRNA to podocytes.

Helpful data on the origin of uEVs were generated by omics analyses on lipid, protein, and RNA composition (1). The proteomic analysis highlighted that 99.96% of proteins present in uEVs mainly originate from the apical part of the urogenital tract following the fluid flux. In fact, the expression of typical apical membrane markers is predominant, even if basolateral markers are also described (18, 19). The remaining fraction (0.04%) of proteins is probably derived from infiltrated cells or from contaminating cells present in the skin (16).

Recently, the contribution of kidney-derived EVs to the entire urinary EV composition was nicely addressed by Blijdorp et al. (12). For this purpose, uEVs isolated from bladder were compared by mass spectrometry to those isolated from a nephrostomy drain. The majority of proteins were in common among the two samples, while only three and 12 proteins were exclusively identified in the nephrostomy and bladder samples, respectively (12). These results confirmed the major contribution of the kidney to the release of uEVs, however, modification of EVs after their release, during the flow, may not be excluded.

Markers typically expressed by uEVs not only reflect the proportion of originating cells but also are characteristic of all the different segments of urogenital tract. The first reports, performed using antibody-based techniques, highlighted the presence of aquaporin (AQP)1, AQP2, cotransporter Na-K-Cl 2 (NKCC2), sodium-hydrogen exchanger 3 (NHE3), and sodium-chloride cotransporter (NCC) (1, 16, 20). Subsequently, thanks to omic approaches, uEVs were extensively characterized. The presence of cubilin, megalin, and aminopeptidase confirmed the release of EVs by proximal tubular cells. The identification of nephrin, podocalyxin, and podocin suggested glomerular and podocyte origins (21, 22). Moreover, bladder and prostate derived-EVs have also been described (18, 23). Some stem cells and regenerative markers such as Klotho, CD133, and stage-specific embryonic antigen-4 (SSEA4) were reported (24–26).

Of interest, novel organelle-containing structures have been described in urine. These membranous structures are released by mechanisms different from conventional exocytosis by proximal tubular cells (27). The extruded vesicles show a size of ∼5 µm, contain entire organelles, and may be found in the EV pellet. In this study, gold nanoparticles were injected into healthy and injured mice and were eliminated by tubular cells through balloon-like extrusions, detected in the urine in a month (27). This phenomenon may have important implications in the elimination of nonbiodegradable nanoparticles and in the self-renew of intracellular organelles for the maintenance of cell homeostasis (27).

Furthermore, uEVs may contain EVs of nonhuman origin such as bacterial or viral EVs. Viruses and EVs have many common characteristics including several mechanisms of action and, in some cases, shape and size (28). Increasing evidences demonstrate that viruses use the machinery of EV packaging and release to deliver viral components (29). Recently, bacterial-derived EVs within uEVs have been reported to approximately reflect the composition of microbiota (30). The five most abundant bacterial phyla are *Bacteroidetes*, *Proteobacteria*, *Actinobacteria*, *Verrucomicrobia*, and *Firmicutes* and their relative EVs have been found abundant both in urine and serum (30). Interestingly, the composition of bacterial EVs within urine may vary based on subjects’ disease status and may be considered a novel source of biomarkers (30–32). In particular, the signature of bacterial EVs present in urine has been analyzed for patients suffering from colorectal cancer and allergic airway diseases observing different patterns for healthy and disease ones (30, 32). Finally, the composition of bacterial EVs in urine of pregnant women, compared with not-pregnant urinary EVs, varies significantly, suggesting a potential role of host-microbe communication and, possibly, of bacterial EVs in pregnancy (33).

## MODULATION OF uEV CARGO AND RELEASE BY PHYSIOLOGICAL AND EXOGENOUS FACTORS

Urine is a fluid that may reflect alterations of not only the urogenital tract but also the entire organism, and, in fact, it has been proposed as source of biomarkers for the prognosis and diagnosis of a wide variety of disorders, including, for example, brain and systemic diseases (34).

It is well known that urine proteome and metabolome can be affected by classical physiological conditions such as hormone status, diet, exercise, daily rhythms, lifestyle, and environments (35). These changes may be reflected in the uEV cargo. Of importance, demographic factors such as age, sex, and ethnicity could also influence EV composition. However, the effects of these factors are, at present, only partially investigated although they are of particular importance for the reliability of the use of uEVs as biomarkers, for further translation into the clinical practice.

A recent study investigated the stability of urinary EV protein content between healthy subjects over a period of 6 mo. Even if the study involved few subjects (8 healthy individuals), results are informative and comprehensive, due to the use of a highly reproducible next-generation proteomic approach (36). The majority of identified proteins displayed high correlation among all samples, presenting 40% of the proteome identified in every sample (36). The study confirms the intra- and interindividual stability of EV proteome (36). This result is in line with previous studies, focused on lower protein numbers, describing a low grade of variability among samples and the presence of a stable protein core (37, 38).

**Figure 2.**
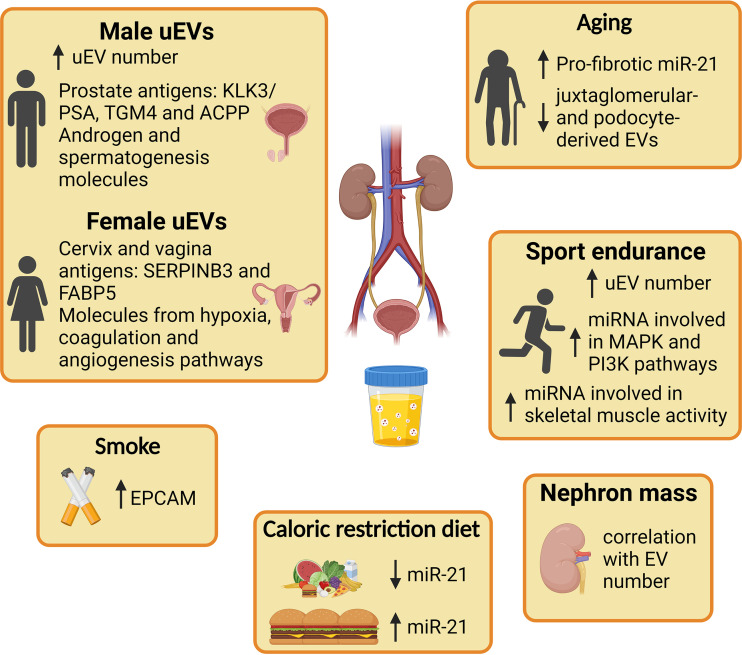
Physiological and exogenous factors that influence uEV cargo and release. Graphical representation of demographic parameters such as sex, nephron mass, and aging that affect uEV composition. Sport endurance, food intake, and smoke are some of the habits that modify uEV cargo. ACPP, prostatic acid phosphatase; EPCAM, epithelial cell adhesion molecule; FABP5, fatty acid-binding protein 5; KLK3/PSA, prostate specific antigen; MAPK, mitogen-activated protein kinase; PI3K, phosphoinositide 3-kinases; SERPINB3, serpin peptidase inhibitor member 3; TGM4, transglutaminase 4; uEV, urinary extracellular vesicle. Created with BioRender.com.

Moreover, the study of Erozenci et al. highlighted a sex-specific signature in uEV proteome. In particular, some proteins [Kallikrein-related peptidase 3 (KLK3)/prostate-specific antigen (PSA), transglutaminase 4 (TGM4), and prostatic acid phosphatase (ACPP)] related to prostate antigens were enriched in man uEVs, whereas Serpin peptidase inhibitor member 3 (SERPINB3) and fatty acid-binding protein 5 (FABP5), associated with cervix and vagina, were increased in female uEVs. Similarly, proteins involved in androgen and spermatogenesis pathways were overrepresented in males. uEVs from females were enriched of proteins involved in hypoxia, coagulation, and angiogenesis, suggesting a connection with the reproductive system (36) (Fig. 2).

Another recent study reported that uEVs excretion is influenced by nephron mass and, consequently, also by sex (12). Specifically, authors found that uEV release correlates with the total kidney volume and since the volume is lower in females than in males, this affects uEV excretion. In particular, the authors collected and analyzed samples of 19 patients before and after a donor nephrectomy and reported a 49% reduction in uEV release in women compared with men. Interestingly, in graft donors undergoing nephrectomy, nephron reduction did not statistically alter uEV levels, probably as a consequence of compensatory hypertrophy. Of interest, a specific reduction of the CD9-positive uEVs was reported (12).

Aging is another factor that may alter uEV content, in line with changes in metabolism and cell activity of the whole body. The overall EV number resulted inversely proportional to age, with a specific decrease of uEVs derived from juxtaglomerular cells and podocytes (39). For instance, the level of miR-21, a miRNA involved in profibrotic and aging signaling pathways, resulted upregulated in uEVs from old rats, as compared with those of young rats (10). Interestingly, caloric restriction prevented this upregulation (10). uEV composition may also be modulated by external factors. Besides demographic parameters, environmental factors and lifestyle, such as hydration status, diet, and exercise, can modulate EV composition and concentration in body fluids (40) (Fig. 2). In human setting, it was reported that miRNA profile of uEVs significantly changes between patients with obesity and healthy subjects and that weight reduction altered uEV miRNA profiles of patients with obesity (41). On the same line, a dietary potassium chloride supplementation affected the uEV level of NCC, proposing uEVs as a tool to assess NCC abundance and activity (42). At variance, levels of Na transporters (NCC and epithelial Na- channel), increased in distal tubular cells due to low Na intake, were found unchanged in uEVs (43). On the contrary, during low-Na diet, the exosomal proteins Alix and CD9 were described significantly increased, without variations in EV number and size (43).

Of interest, several papers highlighted that high-intensity endurance exercise causes an increase of EVs released in blood flow (44–47), while less is known about uEV modulation. In a recent study, Park et al. (48) evaluated the possible effect of high-intensity exercise on uEV release, analyzing uEV samples of healthy men at different time points before and after a run test. uEV concentration was significantly enhanced immediately after the effort, reflecting the increased level of blood-circulating EVs previously observed, but it was rapidly restored to normal values 1 h after rest. Interestingly, uEVs isolated from samples collected immediately after exercise showed significantly increased levels of all three tetraspanins CD9, CD63, and CD81, while 1 h after rest, only CD9-positive uEV subpopulation was maintained at a higher level compared with the basal one (48). Finally, authors performed a miRNA-sequencing analysis aiming to investigate a potential alteration of the uEV miRNA content. Nine miRNAs were modulated by exercise, being involved in MAPK, PI3K-AKT pathways, or related to insulin sensitivity (Fig. 2) (48). Another very recent paper highlights differences in several uEV parameters isolated from inactive subjects and triathlon athletes, studying chronic adaptations of endurance practice (49). uEVs isolated from triathletes resulted in smaller and lower roughness; moreover, miRNAs associated with skeletal muscle activity and metabolic pathways as well as guanosine were differentially expressed by uEVs from the two cohorts of enrolled subjects (49). The study of uEV modulation by sports exercise seems to be of interest by the scientific community with further implications in training, diet of professional athletes, and also in relationship with drugs and doping.

Among additional external factors, cigarette smoke may also alter uEV composition. The effects of exposure to cigarette smoke on EVs present in biofluids such as bronchoalveolar, blood, and urine, were analyzed in a murine model (50). Interestingly, it was reported an alteration in uEV number, size, and epithelial cell adhesion molecule (EPCAM)-positive uEVs by exposure to cigarette smoke (50).

These findings highlight the numerous factors that may impact the uEV signature, including demographic conditions (age, sex, or ethnicity) and personal habits; indeed, further dedicated studies on different factors that may influence uEV number and composition are required to extend findings to the broader population in view of biomarker identification.

## PHYSIOLOGICAL FUNCTIONS OF uEVs

### Role of uEVs in the Intra Nephron Communication

**Figure 3.**
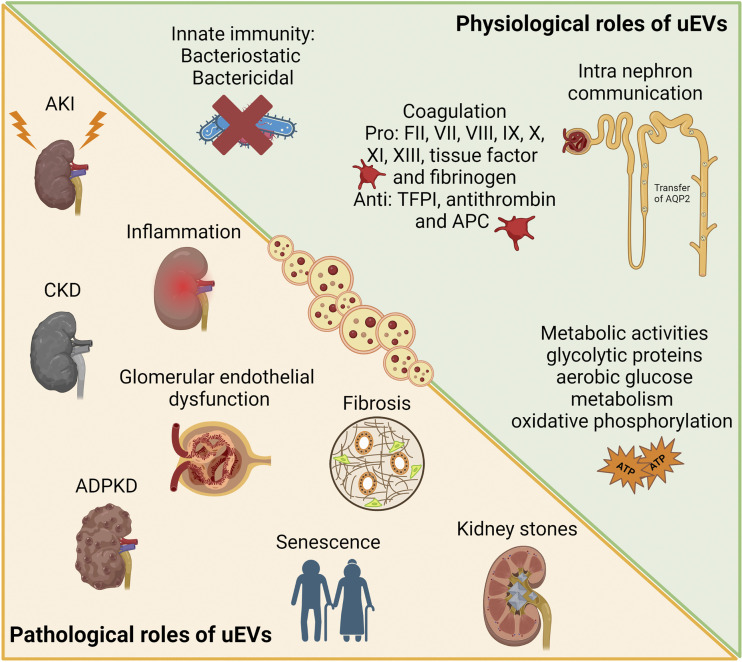
Physiological and pathological roles of uEVs. Graphical representation of different physiological and pathological conditions in which uEVs have been involved in the context of kidney pathophysiology. ADPKD, autosomal dominant polycystic kidney disease; AKI, acute kidney injury; APC, activated protein C; CKD, chronic kidney disease; TFPI, tissue factor pathway inhibitor; uEV, urinary extracellular vesicle. Created with BioRender.com.

uEVs are not only accumulated as waste in the urine but also have an active role in intranephron communication, connecting nephron segments at long distances, following the natural flow of urine (Fig. 3). uEVs secreted by upper segments can be uptaken by downstream cells transferring their message and providing proximal-to-distal signaling and glomerular-to-tubular cross talk. At present, the in vivo demonstration of the uEV-based communication mechanism is still lacking. However, in vitro experiments provide some evidence in support. The first report described the transfer of active and functional AQP2 by EVs released by murine kidney collecting duct cells to recipient cells. Synthetic vasopressin stimulus induced the upregulation of AQP2 within EVs (51). Moreover, a vasopressin analog upregulated EV uptake in tubular cells, while the use of an antagonist reduced this phenomenon (15), suggesting the presence of a regulated mechanism of uEV trafficking. Similar findings showed that EVs released by proximal tubular cells, engineered to express fluorescent exosomal-specific markers CD63 or CD9, could be transferred to different cell lines of the distal tubule and collecting ducts (52). These results support the hypothesis of uEVs’ involvement in the regulation of water and ions transport. Furthermore, stimulation of proximal tubular cells with dopamine agonist induced and increased the production of EVs with an anti-inflammatory activity, reducing levels of reactive oxygen species (ROS) in recipient distal cells (52). Another important aspect in which uEVs may play a role is the intraluminal renin-angiotensin system, being uEV cargo abundant of angiotensin-converting enzyme (53).

However, the in vivo physiological situation appears extremely more complex and can be influenced by the urine composition during physiopathological processes. The presence of flow may modify cell-uEV interaction and the number of particles present in urine may not correspond to the dose used in the in vitro experiments (16). Moreover, the uEV uptake mechanisms in vivo could possibly be modulated by proteins in the uEV corona or by their trapping due to highly abundant proteins such as Tamm-Horsfall (54). Several groups are currently trying to understand the specific mechanisms behind EV uptake and release, modifying or blocking them (55–57). For example, uEVs express, at a high level, CD24 molecule, a small glycosylphosphatidylinositol-anchored protein, which may have a role in EV adhesion to target downstream cells (58). Furthermore, in in vitro experiments, polycystin-1 and polycystin-2 have been proposed to modulate uEV binding of primary cilia of recipient cells (59). Taken together, there are still many open questions regarding the mechanism of EV trafficking within the nephron (60).

### uEVs and Innate Immunity

Of interest, there are evidences of the involvement of uEVs in innate immunity, having bacteriostatic and bactericidal properties (Fig. 3). The anatomic structure of urinary system induces a constant exposure to bacterial infection. uEVs are enriched in molecules implied in host defense and carry proteins that may recognize and bind bacterial external molecules (61). Moreover, it has been described that the presence of uEVs drastically inhibits bacterial growth, favoring their lysis (61). The first involvement of EVs in innate defense was described for airway epithelial cell derived-EVs that expressed surface mucins able to link sialic acid, displaying a neutralizing effect on human influenza virus (62). Interestingly, using a murine model of urinary tract infection, it has recently been described that uEVs are enriched of the iron-binding glycoprotein lactoferrin, mainly released by bladder epithelial cells. Therapeutic administration of EVs containing human lactoferrin significantly reduced bladder bacterial infection and neutrophil infiltration, suggesting an active response by EVs after infection (63). The involvement of uEVs in immunity was recently confirmed by an in-depth proteomic analysis highlighting the presence of about 50% of proteins involved in the glycolytic pathway and in the inflammatory network, known to be the initiator of immune cell response (36).

### uEVs and Coagulation

Another aspect in which uEVs may have a role is in the coagulation process (Fig. 3). The involvement of EVs and, in particular, of plasma-derived EVs in coagulation, is one of the first described roles for EVs, supporting thrombin generation (64). It has also been reported that phosphatidylserine species are present in uEVs and may be supportive of the coagulation process (65). Recently, Saraswat et al. demonstrated the presence in uEVs of several coagulation factors, such as FII, VII, VIII, IX, X, XI, XIII, tissue factor (TF), and fibrinogen. uEVs also express anticoagulant factors, like TF pathway inhibitor, antithrombin, and activated protein C (66). Authors showed an enhancement of thrombin generation by combining uEVs and human plasma, in vitro (66). However, the physiological involvement of uEVs in this process should be further investigated.

### uEVs and Metabolic Activities

Recent proteomic analysis confirmed the presence of metabolic clusters, in particular, glycolytic proteins in uEVs (Fig. 3) (36). These results confirm previous data showing the enrichment in metabolic pathways linked to oxygen consumption and aerobic glucose metabolism (67). Moreover, uEVs were reported to perform oxidative phosphorylation, being able to synthesize ATP and to consume oxygen (68). At present, the real role of these uEVs is still missing and single EV approaches should be performed to better characterize this subpopulation. Authors excluded a potential direct mitochondrial contamination and suggested the presence of mitochondrial inner membrane proteins in the multivesicular bodies, deriving from fusion among mitochondria and endoplasmic reticulum (68). Distinct EV subpopulations, such as small EVs or exomeres should also be assessed (36, 69).

## ROLE OF EVs IN KIDNEY DISEASES

uEVs, thanks to their ability to transport their cargo at long distance, have a central role in the amplification of renal damage (Fig. 3). uEVs contribute in the glomerular-tubular cross talk promoting tubular and glomerular injuries. Moreover, uEVs have been involved in the progression of inflammation and fibrosis, triggering both acute kidney injury (AKI) and chronic kidney disease (CKD) (70).

### uEVs in the Amplification of Acute and Chronic Kidney Injury

AKI is a serious and worldwide renal disorder characterized by a sudden and dramatic loss of kidney function that can eventually progress to chronic injury and to organ failure (70). In the context of tubulointerstitial cross talk, EVs released by the injured epithelium were shown to attract inflammatory cells and stimulate fibroblast-to-myofibroblast differentiation, proliferation, and interstitial matrix deposition, contributing to the extent and persistence of damage and progression toward CKD (71).

EVs released by tubular epithelial cells (TECs) under a proteinuric state expressed the chemokine CCL2 mRNA, which can be internalized by macrophages, leading to increased macrophage migration potential (72). In a subsequent study, it was shown that miR-19b-3p was increased in uEVs from LPS-induced AKI mice, adriamycin-induced CKD mice, and albumin-injured TECs (Table 1). The EV-associated miR-19b-3p might amplify the inflammatory response by promoting M1 macrophage polarization and activating NF-κB signaling (73). Moreover, increased levels of miR-19b-3p were also found in uEVs collected from samples of patients with diabetic nephropathy (73). In a similar way, Li et al. demonstrated that EVs released by TECs maintained under hypoxic conditions increased miRNA-23a expression (74). Like miR-19b-3p, miRNA-23a may induce macrophage switch to M1 phenotype modulating the NF-κB cascade (Table 1). The proinflammatory effect of EVs secreted by hypoxic TECs was confirmed in vivo, as their injection in the renal parenchyma of mice increased the number of inflammatory cells and induced proinflammatory cytokines such as TNF-α, IL-1β, and monocyte chemoattractant protein 1 (MCP-1) clearly suggesting that TEC-derived EVs have a crucial role in the pathogenesis of tubulointerstitial inflammation (74) (Table 1).

**Table 1.** Summary of miRNAs involved in the diseases’ pathogenesis

miRNA	Disease	Type of Model of Study	Description of model of study	Exerted Effect	Mechanism of Action	Reference
miR-19b-3p	AKI and CKD	In vivo	LPS-induced AKI mice	Amplification of inflammatory response by promotion of M1 macrophage polarization and activation of NF-κB signaling	Activation of NF-κB signaling targeting SOCS1	Lv et al. (73)
			Adriamycin-induced CKD mice			
		Human samples	Patients with diabetic nephropathy	Increased levels of miR-19b-3p retrieved in uEVs samples		
miR-23a		In vitro	EVs from TECs under hypoxic conditions	Induction of macrophage switch to M1 phenotype and promotion of inflammation	Modulation of NF-κB cascade targeting ubiquitin editor A20	Li et al. (74)
		In vivo	Injection of hypoxic-TEC-derived EVs in the renal parenchyma of mice	Increased number of inflammatory cells and higher mRNA levels of proinflammatory genes (TNF-α, IL-1β, and MCP-1)		
miR-150		In vitro	Hypoxic proximal TECs	Upregulation of miR-150 expression, direct uptake by fibroblasts in culture		Guan et al. (75)
		In vivo	Ischemia-injured mice	Increase of renal fibrosis		
miR-150-5p		In vitro	Hypoxic proximal TECs	Increased levels of miR-150-5p		Zhou et al. (76)
		In vivo	Unilateral ischemia-reperfusion injury mice	Worsening of renal fibrosis	Inhibition of SOCS1	
miR-21		In vitro	TGF-β1 stimulated TECs	Enrichment of miR-21 levels		Zhao et al. (77)
		In vivo	Unilateral ureteral obstruction mouse model	Increased ECM deposition	Modulation of PTEN/AKT pathway	
miR-221	DKD	In vitro	High-glucose incubated podocyte-derived EVs	Dedifferentiation of proximal TECs	Modulation of Wnt/β-catenin signaling pathway targeting of DKK2	Su et al. (78)
miR-25-3p		In vitro	Podocytes treated with M2 macrophage-derived EVs	Inhibition of apoptosis and EMT induced by high glucose treatment	Inhibition of DUSP1 expression and stimulation of apoptosis	Huang et al. (79)
miR-145		In vitro	TGF-β1-stimulated podocytes	Enrichment of miR-145 levels		Dimuccio et al. (80)
miR-126		In vitro	TGF-β1-stimulated GECs	Reduction of miR-126 levels		Dimuccio et al. (80)

The table summarizes the miRNAs involved in the progression of renal diseases. AKI, acute kidney injury; AKT, protein kinase B; CKD, chronic kidney disease; DKD, diabetic kidney disease; DKK2, Dickkopf-related protein 2; DUSP1, dual specificity protein phosphatase 1; ECM, extracellular matrix; EMT, epithelial-to-mesenchymal transition; EVs, extracellular vesicles; GECs, glomerular endothelial cells; IL-1β, interleukin 1β; LPS, lipopolysaccharide; MCP-1, monocyte chemoattractant protein 1; NF-κB, nuclear factor kappa light-chain enhancer of activated B cells; PTEN, phosphatase and tensin homolog; SOCS1, suppressor of cytokine signaling 1; TECs, tubular epithelial cells; TGF- β1, transforming growth factor β1; TNF-α, tumor necrosis factor α; uEV; Wnt, wingless-related integration site.

Several reports also investigated the profibrotic effect of TEC-released EVs and the possible mechanisms of action. In ischemia-reperfusion-induced AKI mouse model, miRNA profiling of hypoxic proximal TEC-released EVs revealed miR-150 upregulation. In parallel, ischemia-injured mice treated with miR-150-carrying EVs exhibited more pronounced renal fibrosis (Table 1) (75). The presence of miR-150-5p in EVs released by hypoxic TECs and their in vivo profibrotic effect was also demonstrated in unilateral ischemia-reperfusion injury mice (76). On the same line, EVs released by hypoxia-stimulated TECs showed increased level of TGF-β1 mRNA, possibly responsible for fibroblast proliferation and activation (81). In a recent study using CKD mouse models, EVs released by injured TECs were reported to express osteopontin, which through binding of CD44 receptor also promotes interstitial fibroblast activation (82). In parallel studies, TECs isolated from unilateral ureteral obstruction, ischemia-reperfusion injury, or partial nephrectomy in vivo models increased their production of EV-encapsulated Sonic hedgehog proteins that caused activation of interstitial fibroblasts and matrix production. Blockade of EV biogenesis and inhibition of Sonic Hedgehog signaling attenuated renal fibrosis after kidney injury (83). Meanwhile, miRNA sequencing of EVs from TGF-β1-stimulated TECs displayed miR-21 enrichment, that in turn aggravated matrix deposition in unilateral ureteral obstruction mice by modulating the phosphatase and tensin homolog (PTEN)/AKT pathway (Table 1) (77).

Interestingly, EV-mediated progression of renal fibrosis could be due to glomerulo-tubular communication through podocyte EV interaction with TECs. Indeed, EVs from puromycin-injured podocytes have been found to promote apoptosis and synthesis of extracellular matrix proteins (fibronectin and collagen type IV) in cultured TECs through the activation of p38/extracellular signal-regulated kinase (ERK) signaling (84). Similarly, EVs from cultured podocytes could upregulate fibronectin and collagen type IV production of TECs via p38/TGF-β signaling. However, it should be noted that in this study, EVs were derived from untreated podocytes, not from the injured ones (85). These data highlight that injured renal cell-derived EVs are loaded with signaling molecules that can activate profibrotic pathways that promote the initiation and progression of renal fibrosis and CKD.

Interestingly, EVs may also be involved in mechanisms of renal recovery mediated by tubular progenitor cells, a population able to proliferate and promote tissue repair after injury (86). In particular, Zou et al. (87) demonstrated that scattered tubular cell-derived EVs, once uptaken by injured TECs, exerted a beneficial and protective effect and promoted tissue and mitochondrial restoration.

#### Roles of uEVs in diabetic kidney disease.

As for tubular damage, intercellular communication between glomerular cells, i.e., endothelial cells (GECs), podocytes, and mesangial cells, might amplify cell damage and promote fibrosis (Fig. 3). Under high glucose treatment, GECs undergoing endothelial-to-mesenchymal transition secreted EVs rich in TGF-β1 mRNA able to activate the canonical wingless-related integration site (Wnt)/β-catenin signaling in podocytes (88). Such EVs, enriched in TGF-β1 mRNAs, could also be internalized by glomerular mesangial cells, promoting mesangial expansion and matrix overproduction via the TGF-β1/mothers against decapentaplegic homolog 3 (SMAD3) signaling (89). Under high glucose treatment, GEC-derived EVs also contained differentially expressed circRNAs that may limit mesangial cell proliferative ability and promote epithelial-to-mesenchymal transition (EMT). Specifically, downregulated exosomal circRNF169 and circSTRN3 favored the increased expression of the mesenchymal and profibrotic marker α-SMA (90). High glucose-treated glomerular mesangial cells also released TGF-β1-enriched EVs able to induce podocyte apoptosis, reduce matrix adhesion, and downregulate podocyte expression of nephrin, podocin, and Wilms’ tumor-1 (WT1) via PI3K/AKT signaling (91). Finally, EVs originating from high glucose-incubated podocytes have been found to contain elevated levels of miR-221, promoting dedifferentiation of proximal TECs (Table 1). Mechanistically, EV-contained miR-221 directly targets Dickkopf-related protein 2 (DKK2), a suppressor of Wnt/β-catenin signaling, which leads to the acquisition of a dedifferentiated state that is crucial in tubulointerstitial fibrosis (78). Under TGF-β1 stimulation, podocytes, cultured in a millifluidic system with GECs, released EVs enriched of the profibrotic miR-145. On the contrary, EVs shed by GECs were associated with a relevant reduction in the proangiogenic miR-126 (Table 1) (80).

Moreover, EVs released by TECs in the context of diabetes may also mediate tubulointerstitial cross talk, favoring the fibrotic process. In fact, EVs from diabetic mice or high-glucose-treated TECs were potent in inducing the proliferation and activation of fibroblasts. Analyses of the content of proximal TEC-derived EVs suggest that exosomal Enolase 1 could be involved in renal interstitial fibrosis and diabetes manifestations (92). In a different study, EVs from high glucose-stimulated TECs activated the EMT program of neighbor cells in an autocrine manner. Fibulin-1, a glycoprotein known to participate in integrin signaling and in turn triggering EMT, was identified as a possible mechanism (93). On the same line, renal progenitors were also affected by high glucose and albumin treatment, decreasing CD133 levels both in cells and derived EVs (94).

In addition, there were lines of evidence supporting the role of EVs released by macrophages in diabetic nephropathy. In one study, high glucose-induced macrophages were found to shed TGF-β1 mRNA in EVs (95). Another study reported that M2 macrophage-derived EVs containing miR-25-3p prompted a beneficial effect on podocytes by impeding apoptosis and EMT, by inhibiting dual-specificity protein phosphatase 1 (DUSP1) expression and stimulating autophagy (79). Furthermore, in the context of macrophage-macrophage interaction, high glucose-stimulated EVs contained higher levels of IL-1β and inducible nitric oxide synthase (iNOS), leading to the macrophages activation and promoting the expression of inflammatory and profibrotic mediators via the NF-κB signaling pathway (96).

Altogether, these studies corroborate the concept of cross talk among kidney resident cells, wherein EVs are being used as cargo of signaling molecules that can contribute to the establishment and development of diabetic nephropathy.

### Role of uEVs in Kidney Stones

Renal calcification is a complex process that involves the deposition of calcium and phosphate in the renal tissue, leading to the formation of calcified nodules. uEVs have been identified as the mediators of calcification by carrying the calcium-binding S100 family proteins (Fig. 3) (97). Moreover, EVs derived from calcium oxalate-exposed macrophages influenced TECs, by enhancing IL-8 production, as well as activated neutrophil migration and enhanced crystal invasion through extracellular matrix (98). On the other hand, EVs derived from TECs directly treated with calcium oxalate increased oxidative stress and osteogenic changes via MAPK/P-38 pathway (99). Of interest, an in vitro study showed that EVs released by renal tubular brush border membrane allowed faster calcium oxalate nucleation and crystal formation in artificial urine solution (100). This effect was supported by the study of Khan et al. (101) who further demonstrated that crystal deposition in renal papillae might have begun with membrane vesicle-induced nucleation.uEVs also carry dysregulated miRNAs in calcium oxalate stone-forming patients, indicating enrichment of oxidative stress via MAPK signaling pathway and cell adhesion processes via advanced glycation endproducts-receptor for advanced glycation endproducts (AGE-RAGE) signaling pathway (99). These results illustrate the role of uEVs in both initiation of kidney stone formation and induction of proinflammatory and profibrotic processes, which can lead to CKD (102).

### Role of uEVs in Autosomal Dominant Polycystic Kidney Disease

Autosomal dominant polycystic kidney disease (ADPKD) is an inherited cystic pathology consequent to mutations in the multipass transmembrane proteins polycistin-1 and polycistin-2, encoded respectively by *Pkd-1* and *Pkd-2* genes (103, 104). Recent studies suggest that primary cilia-derived EVs have a crucial role in ADPKD (Fig. 3) (105). Specifically, cystic cell-derived EVs and uEVs from patients with ADPKD promoted cyst growth in three-dimensional cultures in vitro and in vivo in a murine model of *Pkd-1* mutant kidneys (105). Moreover, EVs from cystic cells were reported to display a faster uptake by healthy cells and a prolonged half-life, confirming their involvement in cystogenesis occurring in ADPKD (106). In particular, loss of *Pkd-1* promoted cell release of EVs and significantly altered their ζ-potential. This charge difference on EV surface may modify the way EVs interact and bind to target cells, as *Pkd-1*-disrupted cell-derived EVs showed a significantly increased uptake by the kidney (106). Interestingly, Carotti et al. (107) demonstrated a link between *Pkd-1* knockout, increased EV production, and upregulation of ceramide biosynthesis in ADPKD. In particular, reduced levels of polycistin-1 increased ceramides and upregulated ATP signaling, which in turn modulated EV release and favored ADPKD progression.

## CONCLUSIONS AND FUTURE PERSPECTIVES

As discussed earlier, there are several lines of evidence that highlight the relevance of EVs in renal physiology as well as in the progression and amplification of renal inflammation and fibrosis. Cells along the nephron release EVs that may influence downstream recipient cells, carrying their message. Due to their complex cargo, composed by a mixture of proteins, lipids, and different RNA species, uEVs are powerful entities that can modulate the behavior of cells or trigger disease progression, both in neighbor cells or at long distances. As reported earlier, several factors including nephron number, sex, aging, and lifestyle modulate EV release and cargo and could be of interest for better understanding kidney physiology. However, the field has several open questions that deserve further investigations.

On the other side, uEVs appear as an ideal source for biomarkers of renal and urinary tract pathologies and display potential applications, even in systemic diseases. They have the potential to be used in diagnostic procedures with a high level of specificity and sensitivity. The international research community reported recommendations for improving rigor, reproducibility, and best methodological practices to be taken into consideration for preclinical and clinical studies on biomarker discovery (1, 9, 108). In particular, normalization methods and preanalytical procedures should be carefully selected to consolidate data interpretation and results. Altogether, the uEV field has great potential to better understand disease progression and for biomarker discovery.

## GRANTS

This work was funded by the National Institutes of Health (NIH) (1R01DK123234) and is generated within the European Reference Network for Rare Kidney Diseases (ERKNet).

## DISCLOSURES

C.G. and B.B. are members of the Task Force on Urinary Extracellular Vesicles of the ISEV community. None of the other authors has any conflicts of interest, financial or otherwise, to disclose.

## AUTHOR CONTRIBUTIONS

C.G. prepared figures; C.G., A.D., J.J.C., B.S., and B.B. drafted manuscript; C.G. and B.B. edited and revised manuscript; C.G., A.D., J.J.C., B.S., and B.B. approved final version of manuscript.
